# Severe Distal Motor Involvement in a Non-compliant Adult With Biotinidase Deficiency: The Necessity of Life-Long Biotin Therapy

**DOI:** 10.3389/fneur.2020.516799

**Published:** 2020-10-26

**Authors:** Géraldine Van Winckel, Diana Ballhausen, Barry Wolf, Melinda Procter, Rong Mao, Patricie Burda, Davide Strambo, Thierry Kuntzer, Christel Tran

**Affiliations:** ^1^Division of Genetic Medicine, Center for Molecular Diseases, Lausanne University Hospital, Lausanne, Switzerland; ^2^Pediatric Metabolic Disease Unit, Department of Pediatrics, Lausanne University Hospital, Lausanne, Switzerland; ^3^Division of Genetics, Birth Defects and Metabolism, Department of Pediatrics, Ann and Robert H. Lurie, Children's Hospital of Chicago, Chicago, IL, United States; ^4^Department of Research Administration, Henry Ford Hospital, Detroit, MI, United States; ^5^Research and Development, ARUP Laboratories, Salt Lake City, UT, United States; ^6^ARUP Institute for Clinical and Experimental Pathology, University of Utah, Salt Lake City, UT, United States; ^7^Department of Pathology, University of Utah, Salt Lake City, UT, United States; ^8^Division of Metabolism and Children's Research Center, University Children's Hospital, Zurich, Switzerland; ^9^Nerve-Muscle Unit, Department of Clinical Neurosciences, Neurology Service, Lausanne University Hospital, Lausanne, Switzerland

**Keywords:** biotinidase deficiency, spinal cord involvement, tetraparesis, newborn screening non-compliance, biotin

## Abstract

Biotinidase deficiency is an autosomal recessive disorder in which affected individuals are unable to recycle biotin. Untreated, children usually exhibit hypotonia, seizures, ataxia, developmental delay, and/or hearing loss. Individuals diagnosed by newborn screening have an excellent prognosis with life-long biotin supplementation. We report a young adult diagnosed with profound biotinidase deficiency by newborn screening who was asymptomatic while on therapy. At 18 years of age, 6 months after voluntarily discontinuation of biotin, he developed a progressive distal muscle weakness. Molecular analysis of the *BTD* gene showed a pathogenic homozygous duplication c.1372_1373dupT p.(Cys458Leu*fs*Ter26) (1). Despite 16 months since reintroduction of biotin, muscle strength only partially recovered. Transition to adulthood in chronic metabolic diseases is known to be associated with an increased risk for non-compliance. Neurological findings in this adult are similar to those described in others with adult-onset biotinidase deficiency. Long-term prognosis in non-compliant symptomatic adult with biotinidase deficiency likely depends on the delay and/or severity of intervening symptoms until reintroduction of biotin.

## Introduction

Biotin is the coenzyme for multiple carboxylases involved in the catabolism of branched-chain amino acids, the synthesis of fatty acid and gluconeogenesis (2). Biotinidase deficiency (BD) is caused by a defect in biotinidase, the enzyme responsible for biotin recycling (3). The disorder is inherited as an autosomal recessive trait leading to accumulation of toxic metabolites upstream of the enzymatic block that interfere with others metabolic pathways (4, 5). Worldwide, the prevalence of the disease is about 1/60,000 (6). The disorder is classified into profound BD when enzymatic activity is <10% of mean normal serum biotinidase activity and partial BD when residual enzyme activity is between 10 and 30% of mean normal serum activity (4).

Manifestations of BD may develop acutely or insidiously with steady progression or as a series of acute decompensations with intervening normal periods (7). Untreated individuals during infancy or early childhood usually exhibit various neurological symptoms, including hyperventilation, laryngeal stridor, apnea hypotonia, seizure, ataxia, developmental delay and sensorineural hearing loss (8), cutaneous symptoms such as eczematous rash, conjunctivitis and alopecia (9), and/or recurrent infections. The neurological symptoms and the biochemical abnormalities usually rapidly resolve after treatment with pharmacologic doses of biotin, while sensorineural optic atrophy and hearing loss are usually irreversible once they occur (10). Late- or adult-onset complications, such as spinal cord involvement, proximal muscle myopathy or muscle atrophy (8) and loss of visual acuity, have also been reported (11). No clear demarcation exists between when early- and late-onset BD symptoms develops. Individuals may even remain asymptomatic life-long when their residual biotinidase activity is sufficient to recycle biotin and/or in the absence of metabolic stress (10, 12). Life-long oral supplementation with biotin is an easy and cost-effective therapy preventing development of symptoms (8).

We report a young adult diagnosed with BD by newborn screening who voluntarily discontinued biotin supplementation at 18 years old and at 6 months later exhibited severe muscle weakness. This observation should alert medical professionals to consider BD in the differential diagnosis of adolescents and adults with motor limb weakness.

## Case Presentation

**Figure 1:**
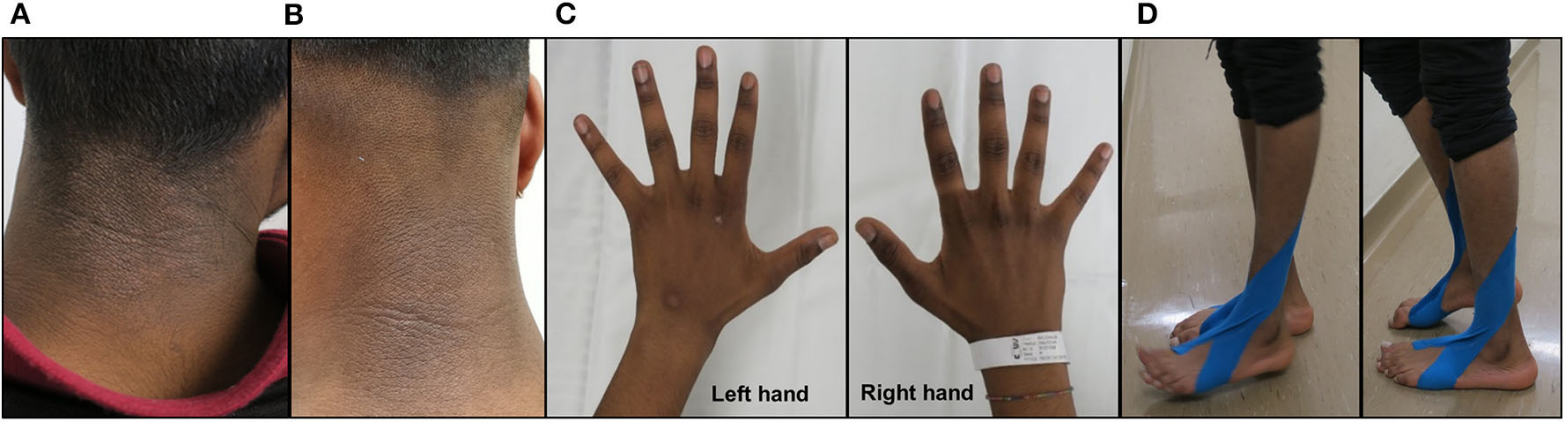
Clinical examination. Eczematous skin rash on the neck prior to biotin supplementation **(A)** and 4 months after biotin supplementation (10 mg/d b.i.d.) **(B)**. Demonstration of muscle wasting of the interossei muscles of the right and left hands prior to biotin supplementation **(C)**. Clinical examination of the individual prior to biotin supplementation showing difficulties in heels (left panel) to toes walking (right panel) **(D)**.

The individual is the oldest of a healthy, three-sibling family of non-consanguineous parents from Sri-Lanka. He was diagnosed with profound BD by newborn screening and treated with 10 mg/d of oral biotin without any complications for 18 years. He had surgery for a sacral meningocele at 2 days of life and for a recurrent dermoid cyst of the conus medullaris at the age of 6 and 9 years. He was regularly seen by pediatric surgeons to follow a catheterization program for a neurogenic bladder due to his sacral meningocoele. During his teenage years, he stopped taking biotin several times. When he started losing his hair, he came to medical attention and was restarted on biotin supplementation. Thereafter, he was lost to medical follow-up because of a difficult social situation. At the age of 18 years, he completely stopped taking biotin supplementation and at about 6 months later, he developed a progressive distal muscle weakness with bilateral foot drop for which he was hospitalized. On physical examination, in addition to wasted distal muscle weakness in the upper limbs and bilateral foot drop, he had partial hair loss and an eczematous skin rash on the neck and left arm (Figure 1). Intrinsic hand muscles were weak (Medical Research Council (MRC) grade 4) and distal foot and toe extensor and flexors were graded MRC 3 to 4. Neither cramps nor fasciculation were seen. Deep tendon reflexes were present and plantar responses were normal. All modalities of sensation, including light touch, vibration, pain and proprioception, were intact bilaterally in the upper and lower limb. He was unable to run and walk on his heels and toes. A mild Gowers' sign was noted.

Brain and spinal magnetic resonance imaging showed no mass effect and no signal abnormality or pathological contrast enhancement on the infra- or supra-tentorial level. No syrinx was seen in the spinal cord. The nerve conduction parameters were within our lower limits of normal values for motor distal latency, conduction velocity and sensory nerve action potential amplitude for median, ulnar, peroneal and sural nerves on both sides. Compound muscle action potential (CMAP) was reduced in amplitude and area following distal stimulation of the median and ulnar nerves and bilateral peroneal nerves (corresponding to 50% of the lower limit of our normal value for median and ulnar and to 10% for peroneal nerves) (13). F-waves were not recorded. No CMAP could be recorded following distal stimulation of the tibial nerves. During voluntary contractions, electromyography showed high amplitudes and reduced pattern recruitment of motor unit potentials in the distal upper and lower limbs muscles. Rare fibrillation potentials were seen at rest. These clinical and electrophysiological findings were consistent with the involvement of lower motor neurons or their motor axons.A biopsy of gastrocnemius muscle showed angulated fibers and fibers type grouping suggesting a neurogenic atrophy.

After admission, the attending metabolic specialist was consulted and suspected neurological complications due to non-compliance of biotin supplementation. Non-compliance over a period of more than 6 months prior to onset of neurological symptoms was confessed when the individual was confronted about taking his biotin, this was confirmed by a serum biotin concentration below 0.1 nmol/L (normal range: 0.3–3.8 nmol/L) in a stored serum sample collected 1 year earlier. Studies performed while he was taking 10 mg/d of biotin showed normal plasma lactate (1.37 mmol/L; normal range: 0.63 and 2.44 mmol/L) and ammonia concentrations (29 μmol/L; normal range: <50 μmol/L). Urinary organic acid analysis disclosed a slight elevation of plasma lactate (197 mmol/mol Cr; normal value <31 mmol/mol Cr). Serum biotin concentration was repeated and showed a normalized concentration of 8.2 nmol/L. Blood count, including mean corpuscular volume and protein electrophoresis were normal, indicating that it unlikely that additional risk factors, such as B9/B12 vitamin deficiency or multiple myeloma, contributed to his symptoms. Given the high suspicion of neurological complications due to non-compliance, biotin supplementation was increased to 10 mg twice a day.

One month after increasing the biotin intake to 20 mg/d, a significant improvement of the handgrip strength measured by Jamar's dynamometer was observed (26.67 vs. 18.33 kg at baseline, resulting in less than the two standard deviations of the normal value). Over the following 3 months, his hair growth improved and his eczematous skin rash disappeared. He recovered muscle strength in the upper limbs and could more easily get up from a squatting position, but the distal lower limbs muscle weakness only slightly improved leaving a persistent bilateral foot drop. Repeated nerve conduction studies showed similar results as seen in the first nerve conduction study, 9 months earlier, with no motor response improvement following stimulation of tibial (no response) and peroneal nerves (CMAP amplitude < 0.1 mV; normal > 2.5 mV). Sensory nerve action potentials were unchanged and of normal amplitudes.

Repeated urinary organic acid analysis showed a normalization of plasma lactate concentration. Serum biotin concentration increased to 70 nmol/L. Because the biotinidase activity measured at the time of the initial diagnosis was not available, it was repeated at the Department of Metabolic Diseases, Children's Hospital Zurich, Switzerland and was completely absent in serum confirming profound BD. Molecular analysis of the *BTD* gene was performed at the ARUP Institute for Clinical and Experimental Pathology, University of Utah, USA, and revealed the known pathogenic, homozygous duplication c.1372_1373dupT p.(Cys458Leu*fs*Ter26), resulting in protein truncation (1).

## Discussion

Our individual with profound biotinidase deficiency was identified to have the disorder by newborn screening. His molecular analysis indicates that he is homozygous for two identical frameshift mutations that result in a severely truncated enzyme protein with the total absence of biotinidase activity. When he was younger and discontinued biotin, he developed symptoms that are more typical of those seen in untreated children with the disorder. However, at 18 years of age, when he discontinued biotin, he not only developed some hair loss and a skin rash, but more importantly, he developed the neurological symptoms that are more commonly observed in other adolescence and adults with untreated biotinidase deficiency (11). Of interest is that this individual exhibited major symptoms after at least 6 months of biotin discontinuance. One cannot predict how long after biotin abstinence symptoms will occur in a child or adult with profound biotinidase deficiency. We are aware of adults shown to have profound biotinidase deficiency who have remained asymptomatic without supplemental biotin (14, 15). It is possible that later onset of symptoms in these individuals, as well as ours, may be due to an unknown or unintentional increase in dietary intake of free biotin, which seem unlikely in our case, or to as yet unknown epigenetic effects.

Only a few reports detail the outcome of individuals diagnosed with BD by newborn screening who discontinue their biotin supplementation. The time until onset of symptoms after treatment interruption is variable among older individuals, but it may take several months or even years to appear. In our case, the period of biotin cessation was likely more than 6 months. Reintroduction of biotin at the same or higher doses usually improves most symptoms, unless too long a period has elapsed since the onset of symptoms (4, 16, 17); this can result in irreversible neuronal damages.

Neurological presentation appears to be different in childhood and in adulthood. Brain involvement seems to be more common in the early-onset form and involvement of spinal cord, optic and peripheral nerves in the late-onset form. This may potentially be explained by the susceptibility of specific brain areas to maturational or metabolic factors that decrease with age, whereas other regions, such as spinal cord and optic nerves, are still at risk (18, 19). Given that our individual had good recovery of strength in his upper limbs only with the biotin therapy, we cannot exclude that the lack of complete response of the lower limbs to the reintroduction biotin may be related to his prior spinal cord abnormality (i.e., corrected sacral meningocoele, past recurrent dermoid cysts of the conus medullaris and ongoing catheterization program (all of which are not usual findings in individuals with biotinidase deficiency) with potential vulnerability of the lower motor neurons (20, 21).

Late-onset manifestations in BD have been described in individuals before newborn screening was available and currently in countries where newborn screening is still not implemented. Several individuals between the age of 22 months and 22 years have been reported with spinal cord involvement as initial manifestations (5, 7, 11, 18, 22–33). This complication seems to affect predominantly children or young adults with profound BD, often triggered by intercurrent illness, and manifests as spastic tetra-, or more frequently, paraparesis. In all cases, there was improvement after several months with various doses of biotin (10–300 mg/d), but mild paresis of lower limbs or residual spasticity may persist, as in our individual.

Neurological manifestations are a leading hallmark in BD. This may be explained by the fact that biotinidase activity is relatively low in brain compared to that in other tissues and is localized only in certain regions of the central nervous system (red nucleus, cerebellar Purkinje cells, lower auditory brainstem nuclei) (6). Therefore, the brain likely cannot readily recycle biotin and is dependent on biotin transferred across the blood-brain barrier, making this organ more vulnerable to biotin deprivation.

Our individual developed peripheral neuropathy or lower motor neuron syndrome after discontinuing biotin therapy. This situation illustrates that the transition process from childhood to adulthood is a crucial step in the follow-up of individuals affected by chronic metabolic diseases who are in need of regular medication. Non-compliance may occur more often in enzyme-deficient individuals who do well-clinically and do not have periodic follow-up evaluations as adolescents or young adults.

We would also like to highlight the diversity and non-specificity of neurological manifestations in individuals with untreated BD, whether or not identified by newborn screening (5). Spinal cord involvement with muscle weakness in a young adult who was not identified by newborn screening or who is not compliant with biotin therapy must alert medical professionals to perform metabolic investigations. Any delay in diagnosis and initiation or reintroduction of treatment can lead to life-long, avoidable, irreversible disabilities (25, 34). The prognosis appears to depend on the delay between appearance of symptoms and introduction of biotin (7, 12). Biotin effectively alleviates most symptoms, but its efficacy depends not only on the time the individual is symptomatic, but also on the severity of the symptoms (8). Even though BD seems to be an easily treatable disease, it is of utmost importance to follow regularly individuals with this inborn error of metabolism and/or to assure that their families are aware of symptoms and complications of non-compliance. Biotin is an indispensable life-long therapy for individuals affected by BD.

## Ethics Statement

Ethical review and approval was not required for the study on human participants in accordance with the local legislation and institutional requirements. The patients/participants provided their written informed consent to participate in this study. Written informed consent was obtained from the individual(s) for the publication of any potentially identifiable images or data included in this article.

## Author Contributions

GV, CT, TK, DB, and BW contributed to conceptualizing, drafting, and revising the study. TK and DS contributed to analyzing and interpreting the ENMG data. CT, TK, GV, and DS contributed to acquiring and the interpreting of the clinical information. BW, MP, and RM contributed to analyzing of molecular data. PB contributed to analyzing of enzymatic data. All authors contributed to the article and approved the submitted version.

## Conflict of Interest

The authors declare that the research was conducted in the absence of any commercial or financial relationships that could be construed as a potential conflict of interest.
